# Membranous Formations on Mucous Surfaces

**Published:** 1872-07

**Authors:** Frank A. Ramsey

**Affiliations:** Memphis, Tennessee


### MEMBRANOUS FORMATIONS ON MUCOUS SURFACES.

BY FRANK A. RAMSEY, M.D., MEMPHIS, TENNESSEE.

[Read to the Clinical Society of Memphis, at its Session, Tuesday, April 30, 1872.]

Your attention, gentlemen, having been completely filled at the last session of the Clinical Society, by a member exhaustively considering the subject of pleuritic effusion, it seems to me to be somewhat in keeping, and opportune, to present to you a membranous-like formation, that was dejected by a patient at present under my professional administration, and to make it the occasion of some remarks. I can only regret the inability of your present reporter to give his subject the same clearness in expose and completeness in detail that so recently attached here to the subject with which I have assumed it to have some connection.

Serous effusions, resulting from inflammation, are much more common than membranous formations on mucous surfaces, resulting from inflammation. They probably, however, each indicate life-power perverted in the direction of depression. In inflammation of the serous tissues, except the life-force is inadequate, proliferation and adhesion occur; if inadequate, proliferation and capillary emission of fluid succeed. In mucous inflammation, except the life-force is inadequate, the exhaling or proper vessels of the surface, and the mucous follicles, pour out fluids; and if inadequate, a coagulable albuminous matter is furnished, and concretes in the form of a false membrane on the surface that produced it. Properly minute examination exposes the fact that such formations do not have any trace of fibres, laminæ, canals or areolæ, and hence are said to be apparently organized, and dependent upon the adjacent tissues for that vitality, though there are respectable investigators who affirm organization, of admitted low degree, to be established in the occurrence of concretion in the form of membrane.

The specimen which I present to you, when dejected, was regularly cylindrical, and preserved that shape through several successive pourings of water upon it, and until it had been for a considerable time in the vial that now contains it, and in which it has been subjected to shaking by those who have examined it curiously. It was passed by an educated female, who lives in an easy pecuniary condition, with pleasant social surroundings, and in happy domestic relationships. She is forty years of age, and has been delivered at full term of half a dozen children; perineal rupture occurred at first labor. She says that for seventeen years she has never known a moment that she was free from pain. Pain is not now, and I can not learn that it ever was, fixed. Of course she is hysterical, multitudinously so; but, withal, she is reasonable, and is abundantly profuse in acknowledging the leniency and patience of her husband, family, and acquaintances, with her variableness of temper and deportment, and constant complainings.

For many years, she has taken food more from a sense of duty than from desire, and without relish—always but little at a time, and in the aggregate not enongh to accomplish more than just to prevent her from dying by starvation. She is anæmic She was the subject of corporal metritis, with uterine hemorrhage—for which she was treated by appliances to the affected part, so judiciously selected and skillfully administered as to have effected restitution locally to the fullest degree permitted by the depraved condition of her whole economy. She now menstruates regularly; and for a few days before, and for a few days after the eruption, she has profuse leucorrhœa, which, though it is constant, is much less abundant at other times, and than formerly. She has had, and has, hemorrhoids and rectitis. This last, occasionally, is very positive; the sub-acute or chronic action, at such times, judging by local and general symptoms, is intensified. She has had, and recently, fissured anus; and all the while has given evidence of the existence of a catarrhal condition of the bowel. Ordinarily, constipation prevails; but she has presented occasions when treatment for diarrhœa and for dysentery was necessary. She is relatively sleepless, and is always anxious for company, and when she has it, is exceeding voluble, and seemingly felicitous; but when alone, she is depressed in spirits, and profoundly so on the approach of, during, and immediately after, menstruation.

She had been confined to bed for seven weeks when I made my first call, and during that time, she took regularly every night fifteen or twenty grains of chloral, contrary to the advice of her physician, by whom it had been at first necessarily given, because of the inadequacy of other agents to occasion sleep. During these weeks, she complained all the time of a burning in the rectum, and frequently mentioned that she had passed shreds of her bowel, and at such times affirmed that the burning, though not destroyed, was modified, until ultimately the shreds, at one stool, were passed in heavy quantity, and with them several inches of the same membranous substance, but in a well-formed cylindrical shape, and immediately the burning sensation ceased, and has not yet, again, troubled her. Under a strict observance of the advice not to take any nervous medicaments except whisky, and a systematized administration of nutrients at short intervals and at designated moments, she has improved in strength, and has accreted tissue, but is yet very far from having attained a condition of health.

There have been, relatively, but few observers who have made record of the passage of pseudo-membranes from the bowels; but the few are uniform in affirming it to have occurred only in instances of prolonged ailment, and, almost without exception, in female patients. They concur, too, in symptomatology—representing the bowels as irregular in action, either too relaxed or too costive; and after intervals of great or less duration, simple uneasiness, curiously located pains and sensations, or sometimes even violent colic, will be felt; and under the operation of a purgative agent, or a spontaneous laxness of the bowels, the stools contain portions of the false membrane, which continue to be voided for several days. The shreds vary in size, and occasionally form into complete tubes of considerable length, and there is marked subsidence of symptoms, which do not recur until the morbid formation is again developed and begins to be detached. These are described as occasionally white and soft, and sometimes yellowish, consistent, and even elastic; and are said to be produced in any part of the intestinal canal, or in both the small and large bowels at the same time. Instances are on record in which the membrane was discharged from the uterus and the vagina almost contemporaneous with its discharge from the bowels.

These membranous formations upon the mucous surface of the intestines were considered by Dr. Powell, who first described them, to have been formed in a similar manner to those observed in croup, and, in a few instances, in bronchitis. They are said to occur with much greater frequency in the mouth, pharynx, and œsophagus, than at other points, and presents then in the form of whitish-flocculent or thin membranous-like patches and shreds, covering the inflamed surfacce, occurring in the course of severe or protracted febrile disorders, particularly so in scarlet fever, and is the characteristic attendant of an epidemical scourge with which we are all familiar.

The constitutional debility and depression of spirits connected with protracted ailment are especially concerned in occasioning the peculiar condition of the vessels and tissue involved in the production of this matter; and the occurrence of it in the course of more rapid affections is indicative of a peculiar failure of life-power. This is the clinical interest that attaches to the formation of membranes on mucous surfaces under any circumstances; the life-power is depressed—the vital force, whatever that is, impaired, and clearly pointing to the necessity of such agents as will do most, under the circumstances of individual cases, toward putting the channels and instruments of its play in a condition conducive to the exercise of its proper power. These agents can not be definitely determined, except by the judgment of the practitioner when exercised upon an individual case. If any circumstance prevails that readily, or upon investigation, is demonstrable as contributing to debility, though it may have no other manifestation than the membranous formation, that circumstance ought to be destroyed, or the patient ought to be relieved of its influence. If sleepnessness prevail, sleep ought to be, if possible, induced; but if the means employed for that end become contributing sources to the debility, they ought to be put away—for in administering, we must always keep in mind, that agents possessed of curative power may be potent causes of disease.

When the membranous formation is enteric, it becomes a point of some delicacy to determine the propriety of giving the patients purgative medicaments. In inflammation of the stomach and in inflammation of the bowels, separately or together, when acute, reason and observation concur in condemning resort to any agent at all capable of disturbing the rest which we desire the tissues to have. But in some instances, the presence of hard fæcal matter is promotive of the diseased action of the vessels and tissues with which it is in contact, and the practitioner must decide a question that inevitably will arise in every case, whether acute, sub-acute, or chronic, of inflammation of the first passages.

In the case reported here, an occasional purge has been found to be absolutely necessary. Each time of administration has been the occasion of some one or another intensified symptom—has been of temporary disadvantage; but subsequently, perhaps, the condition of the patient has been improved by the purgative action.

One point more, and I am done. Notwithstanding the fact that depression of life-power is indexed by the occurrence of pseudo-membranes on mucous surfaces, there are occasions when its appearance may be hailed as evidencing a return, in successive steps, of the tissues to the normal or healthy condition.

Sanderson, in his remarks appended to Simon's treatise on the subject, in Holmes' Surgery, gives us to understand, that amongst the first phenomena in inflammation, is the return of the involved tissue from a state formed, to a plastic, or forming condition, and so on successively until the end in the particular instance; and that, in the final attainment of health, these steps are passed in reversed succession. I refer to this teaching to sustain me in the clinical position I have just taken—to sustain me in sometimes regarding the appearance of pseudo-membranes on mucous surfaces as sufficient to essentially mollify an otherwise very unfavorable opinion of a case of disease in progress. I could cite instances to support me that have occurred to myself in practice, but I prefer one that is on record, and that can be consulted by any one who may elect to give the point consideration.

In the April number, 1868, of the *New-Orleans Journal of Medicine*, is recorded "Observations on Diphtheria," by Dr. H. D. Schmidt, of that city. The observations are based upon one case of diphtheritic membrane, occurring under circumstances that fit it for my purpose here.

The patient was a girl, æt. seven years, living under cosmic circumstances of the most deleterious kind, in a hot climate, on a ridge surrounded by marshy lands and bogs, and very near a particular bayou, the water of which has a closer analogy to the liquid of a cess-pool than to the water from an inlet to the ocean. The patient first sickened with yellow fever, from which she recovered in a little more than a week, terminating about the 12th of September. On the 30th of October, she was again placed under the observation of the practitioner of medicine, having been subject to repeated attacks of chill and fever for a week, and now had very high fever, though her appearance was very pale and anæmic, and she was troubled with cough. During the next ten days, fever of remittent type prevailed with her; she had moist, yellowish-white-furred and slightly swollen tongue; cough; loose bowels, easily checked by bismuth, mucilage and paregoric; pain in epigastric region, below the sternum and extending to hypochondrium, that was relieved by a small blister over her chest; very much disposition to sleep. No improvement for four days, when, at a morning visit, the tongue was dry, covered with a yellowish-brown scab, and had several fissures; purplish gums, slightly tumid, sordes; feeble pulse, about one hundred to the minute; no epigastric, but umbilical pain, and swollen and tympanitic abdomen. Typhoid fever now diagnosed. Oil of turpentine, with camphor, was administered, and was followed, as observed next morning, with moist tongue, a little swollen, and covered with a white, puslike secretion, with a smooth surface beneath it. This was regarded as favorable, though the disposition to sleep, and apathy, were marked, and tympanitis was positive, and abdominal pain was increased, and diarrhœa was active, and cough was troublesome. About the second day after the tongue had become moist, a white exudate appeared back upon the hard palate, near the place where it is joined to the soft palate. From here it spread, in the course of a few days, over the whole mucous membrane of the oral cavity, including the fauces, and to the very edge of the lips; from the lips to the posterior arches, there was no place free from it, except the margin of the gums. The observer here says, "The disease had now thrown off its last mask, and stood there in the form of diphtheria." For a week, every typhoid symptom intensified, and before the end, rheumatism and hemorrhage occurred, and the urine contained albumen. During the passing of time, the exudate, under applications, became gradually loosened at points, so that it could be taken away in patches; but while this happened in one place, it extended in another.

From the first, supporting diet had been given, and under the use of chlorate of potassæ, the stupor disappeared in the most striking manner, the change in the cerebral functions being next to the occurrence of false membrane as an indication of favorable progress. Other typhoid symptoms continued until impacted fæces were removed under the action of a purgative agent; and then the progress of the case was onward, until the patient enjoyed better health than she had ever done.

---

Tartrate of Iron and Potassa in Typhoid Fever.—Dr. Charles H. Gordon, Mokelumne, California (*Pacific Medical and Surgical Journal*), calls attention to the use of tartrate of iron and potassa in typhoid fever—the ulceration of the intestines in typhoid being modified by its employment. He usually prescribes, for an adult, five-grain doses in solution twice a day, increasing the dose in the second and third week to double that quantity.—*The Medical Record.*

